# *Wolbachia*-induced cytoplasmic incompatibility triggers intergenerational dysregulation of the small RNA regulatory network in offspring

**DOI:** 10.3389/fmicb.2026.1764569

**Published:** 2026-03-05

**Authors:** Weihao Dou, Tianchu Li

**Affiliations:** 1 School of Life Sciences and Medicine, Shandong University of Technology, Zibo, Shandong, China; 2 Translational Medicine Center, Zibo Central Hospital, Zibo, Shandong, China

**Keywords:** cytoplasmic incompatibility, immune, miRNA, piRNA, *Wolbachia*

## Abstract

The intracellular symbiont *Wolbachia*, which is widespread among insects, may induce cytoplasmic incompatibility (CI) between hosts with different infection statuses. Increasing evidence indicates that symbiotic bacteria can influence host reproduction, metabolism, and other biological processes by modulating non-coding small RNAs. However, it is still unclear how *Wolbachia*-induced CI affects the offspring reproduction. In this study, using *Drosophila melanogaster* as a model system, small RNA and transcriptome sequencing were conducted on the reproductive systems of the offspring resulting from crosses between *Wolbachia*-infected males and uninfected females. By comparing F1 males and females to their respective paternal or maternal lines, we identified distinct intergenerational discrepancies. The male offspring of the CI cross showed a significant upregulation of immune-related genes and a notable downregulation of reproductive-related genes. Moreover, the microRNA regulatory network in the testes of the offspring was significantly disrupted, with the target genes directly involved in embryonic development, energy metabolism, immune regulation, and reproductive behavior. Additionally, increased transposable element (TE) expression and piRNA dysregulation were observed in the testes of male offspring. Overall, this study offered new insights into the intergenerational regulatory effects of *Wolbachia*-induced CI and its potential mechanisms.

## Introduction

*Wolbachia* is an intracellular endosymbiotic bacterium widely distributed among arthropods and nematodes, infecting approximately 40–65% of arthropod species worldwide (Weinert et al., 2015). This bacterium manipulates host reproductive systems to maximize its own transmission, with cytoplasmic incompatibility (CI) being the most prominent mechanism. CI is a phenomenon in which matings between *Wolbachia*-infected males and uninfected females (or females infected with a different *Wolbachia* strain) result in embryonic lethality or a strong female-biased sex ratio. This reproductive manipulation strategies enable *Wolbachia* to rapidly spread within host populations (Ross et al., 2019). In addition to regulation of reproduction, *Wolbachia* has also been shown to affect host metabolism, immune responses, and lifespan. Moreover, in some insect species, it even confers antiviral or antiparasitic protection (Chrostek et al., 2021; Zhu Y. X. et al., 2024; Lindsey et al., 2018; Osorio et al., 2023). Currently, *Wolbachia*-induced CI has demonstrated great potential for application in mosquito and vector-borne disease control (Liang et al., 2024; Montenegro et al., 2024).

The molecular basis of CI is traditionally explained by the “modification–rescue” (mod–resc) model, which proposes that one factor expressed by *Wolbachia* modifies sperm, and another factor expressed by *Wolbachia* in the egg can rescue this modification (Werren, 1997). Recently, the *Wolbachia* genes responsible for CI induction and rescue have been identified (Beckmann et al., 2017; LePage et al., 2017). These genes are organized as an operon-like genetic element. In *Drosophila melanogaster*, the CifB protein acts as the CI-inducing factor, whereas CifA mediates CI rescue, consistent with the “mod-resc” model (Shropshire et al., 2020a,b). CI intensity in *D. melanogaster* is highly variable. CI intensity is highest in young (1-day) males (nearly 100%) and declines rapidly with male age and successive matings, independent of *Wolbachia* density or *cif* gene expression (Shropshire et al., 2021; Yamada et al., 2007). This suggests that host factors play a crucial role in *Wolbachia*-induced CI. With the advancement of multi-omics technologies, researchers have conducted comprehensive omics studies on *Wolbachia*-induced CI, revealing that *Wolbachia* infection can cause dysregulation of multiple metabolic pathways in the host reproductive system, particularly those related to reproduction and immunity (Zheng et al., 2011b; Dou et al., 2021; Zheng et al., 2011a; Biwot et al., 2020; Xi et al., 2008).

In recent years, the roles of non-coding small RNAs (sRNAs), particularly microRNAs (miRNAs) and PIWI-interacting RNAs (piRNAs), in reproductive regulation and epigenetic modification have garnered increasing attention (Leal-Galvan et al., 2024; Ren et al., 2023; Kumar et al., 2025). miRNAs regulate gene expression at the post-transcriptional level and are involved in key processes such as germ cell differentiation, gamete maturation, and embryonic development (Song et al., 2024; Yan et al., 2024). In *Aedes aegypti, Wolbachia* has been shown to modulate miRNA expression, thereby affecting its intracellular density within host cells (Osei-Amo et al., 2012). In *Tetranychus urticae, Wolbachia* infection significantly suppresses miRNA expression, and the predicted target genes of these miRNAs are enriched in lysosomal function, apoptosis, and female reproduction pathways (Rong et al., 2014). Similarly, in *Laodelphax striatellus, Wolbachia* infection triggers sex-specific differences in miRNA expression. These miRNA targets are enriched in reproductive and immune pathways, linking *Wolbachia*-induced disruption of small RNA networks to male infertility (Liu et al., 2019). Collectively, these findings highlight a perturbation of host small RNA-mediated regulation as a key factor in *Wolbachia*-induced CI.

Recent research has also shown that sRNA regulatory networks in reproductive tissues play an important role in transgenerational epigenetic regulation. For example, in nematodes, sRNAs shuttling between cells and tissues can transmit information to the germline, promoting longer lifespan and stronger stress resistance in offspring (Zhang and Tian, 2022). In locust reproductive systems, miRNA can produce epigenetic regulation and cause transgenerational effects in offspring (Zhu Y. N. et al., 2024). However, current research focuses on *Wolbachia*'s regulation of host reproductive capacity, and whether *Wolbachia*-induced CI has intergenerational effects remains unclear.

In this study, we compared progeny from CI crosses (*Wolbachia*-infected males × uninfected females) with their parental controls using combined transcriptome and small RNA sequencing in *D. melanogaster*. Our analyses revealed sex-biased differences in both gene expression and small RNA regulatory networks in the hybrid offspring. Furthermore, the data suggest that CI may impair male reproductive capacity in offspring by disrupting these small RNA-mediated regulatory networks. Our findings provided new insights into the dynamic interactions between hosts and their symbionts, laying the groundwork for further research into the molecular mechanisms underlying insect reproductive regulation and population evolution.

## Materials and methods

### Fly strains and culture conditions

The *D. melanogaster* strain used in this study was kindly provided by Professor Haoyuan Hu. *D. melanogaster* naturally infected *Wolbachia* strain *w*Mel in our laboratory (designated as the W population), with an initial population size of around 200 individuals. To acclimate the flies to laboratory conditions and homogenize their genetic background, the W population was maintained on a standard cornmeal medium (cornmeal 60 g, brown sugar 30 g, sucrose 30 g, agar 8 g, sodium benzoate 2 g, yeast powder 10 g, water 800 mL) for five generations, starting in 2018. The population size remained stable at around 1,000 individuals throughout this period.

The establishment of the *Wolbachia*-free population (designated as the T population) has been described previously (Dou et al., 2023). Briefly, the W population was treated with tetracycline for three generations to eliminate *Wolbachia*. To restore the natural gut microbiota and eliminate potential antibiotic effects, the T population was subsequently reared on standard cornmeal medium supplemented with feces from the W population.

### Mating experiments and RNA extraction

**Figure 1:**
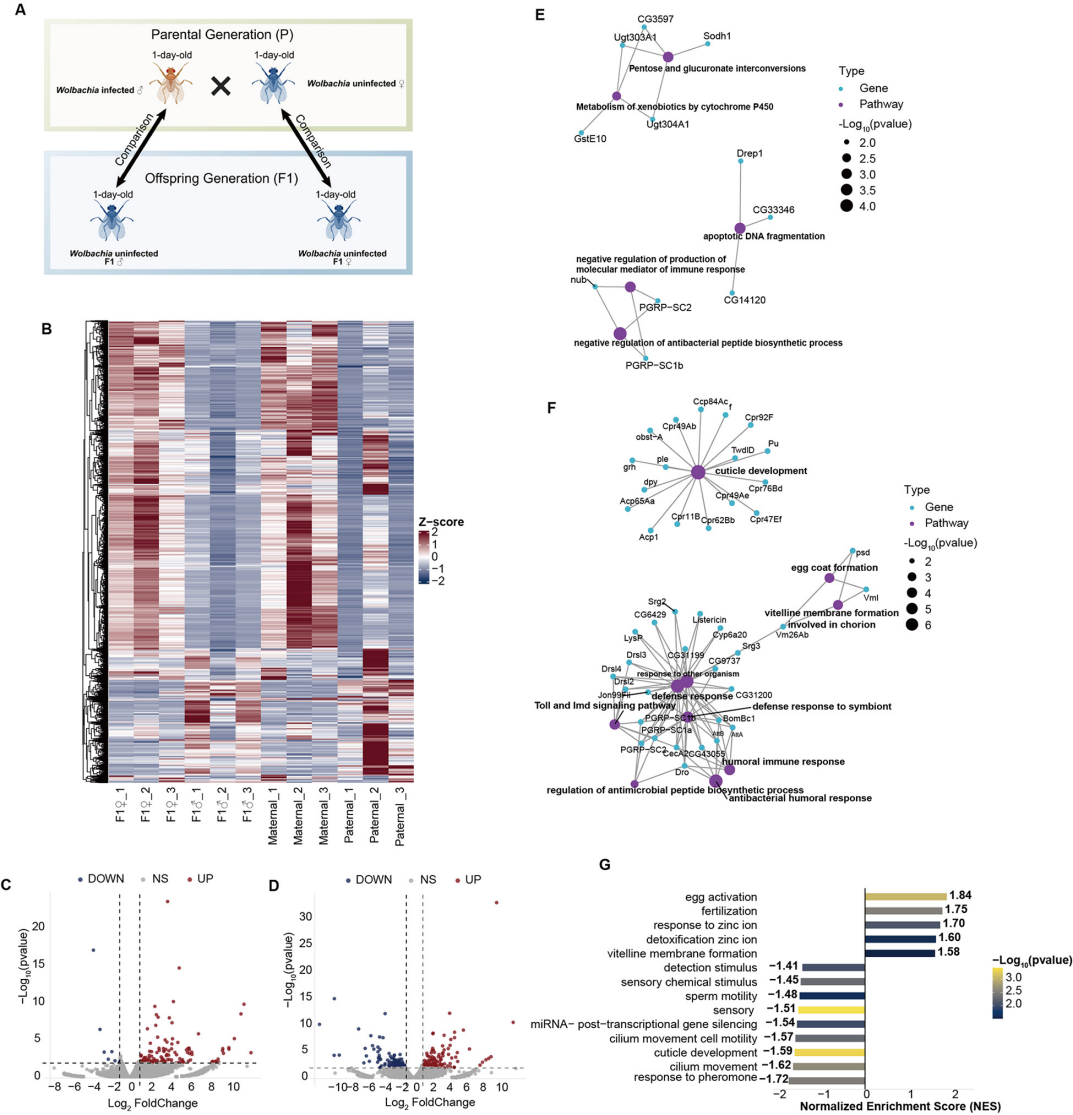
Differences in gene expression within the reproductive system of F1 offspring resulting from crosses between *Wolbachia*-infected males and *Wolbachia*-uninfected females, compared to their parental lines. **(A)** Schematic illustration of the experimental design and comparative analysis. **(B)** Relative gene expression across all samples. Gene expression levels were normalized using *Z*-scores based on TPM values across all samples. **(C)** Volcano plot of DEGs between F1 females and maternal lines. **(D)** Volcano plot of DEGs between F1 males and paternal lines. **(E)** GO enrichment network of DEGs in females. Purple nodes represent GO terms, and connected nodes indicate the genes included in each term. **(F)** GO enrichment network of DEGs in males. **(G)** GSEA enrichment analysis of all genes in males.

One-day-old *Wolbachia*-uninfected virgin females were crossed with *Wolbachia*-infected males [Figure 1A; The figure was created with BioGDP.com (Jiang et al., 2024)]. To obtain sufficient offspring, 20 females and 20 males were placed together for mating. After 24 h, males were removed, and females were allowed to lay eggs for 24 h before being discarded. Once the F1 progeny emerged, adult flies were immediately collected, and males and females were separated to prevent mating.

Testes and ovaries were dissected from representative individuals of the parental generation and their corresponding F1 progeny. To eliminate the influence of developmental time, we selected 1-day-old virgin female and male parents (*Wolbachia*-infected males and *Wolbachia-*uninfected females) for dissecting reproductive tissues for RNA extraction. Similarly, for the offspring produced by the hybridization, 1-day-old virgin female and male individuals with morphologically normal were selected, dissecting reproductive tissues for RNA extraction. To prevent RNA degradation, flies were anesthetized on ice, and tissues were dissected in cold PBS (137 mM NaCl, 2.7 mM KCl, 10 mM Na_2_HPO4, and 2 mM KH_2_PO4; pH 7.4; pH 7.4). Approximately 20 flies were pooled into a single biological sample, with three biological replicates per group. Dissected reproductive tissues were immediately transferred into TRIzol reagent (TransGen, Beijing, China) and stored at −80 °C until further processing. RNA extraction was performed according to the manufacturer's protocol for the TransZol Up Plus RNA Kit (TransGen). The extracted RNA was dissolved in RNase-free water and stored at −80 °C.

RNA concentration and purity were measured using a NanoDrop One spectrophotometer (Thermo Fisher Scientific, Waltham, MA, USA), and RNA integrity was assessed through agarose gel electrophoresis. Qualified samples were subjected to Illumina PE 150 bp library construction and sequencing on the HiSeq 2500 platform (Illumina, San Diego, CA, USA). For small RNA sequencing, libraries were prepared using the NEBNext^®^ Multiplex Small RNA Library Prep Set for Illumina^®^ (NEB, E7300L). Total RNA served as input, with adapters ligated to both ends of the small RNAs. This was followed by reverse transcription to produce cDNA, and paired-end 50 bp sequencing was performed on the Illumina HiSeq 2500 platform.

### Bioinformatics analysis

#### Transcriptome analysis

Raw RNA-seq reads were first processed for quality control and adapter trimming using fastp v1.0 (Chen et al., 2018) to remove low-quality sequences and adapter contamination, with these parameters: fastp-q 20-u 10-n 5. Then, high-quality reads were aligned to the *D. melanogaster* reference genome using HISAT2 v2.2 (Kim et al., 2019), and the resulting alignment files were converted and sorted with samtools v1.18 (Li et al., 2009). Expression levels were quantified using featureCounts v2.0 (Liao et al., 2014), which generated a raw read count matrix for each sample.

Due to the significant biological dimorphism between testes and ovaries, male and female datasets were analyzed separately to identify generation-dependent changes within each sex. Differential expression analysis was carried out using DESeq2 v1.12 (Love et al., 2014) to identify differentially expressed genes (DEGs) based on the criteria of |log_2_FC| ≥ 1 and raw *p-*value < 0.01. We selected this threshold to minimize the exclusion of biologically relevant genes (false negatives) potentially caused by strict FDR correction; the reliability of these candidates was subsequently confirmed by qPCR validation. Functional enrichment of DEGs was performed with clusterProfiler v3.18 (Yu et al., 2012), including Gene Ontology (GO) and Kyoto Encyclopedia of Genes and Genomes (KEGG) pathway analyses to reveal enriched biological processes and molecular functions. Additionally, Gene Set Enrichment Analysis (GSEA) was employed to determine whether predefined gene sets exhibited significant enrichment trends. Results were visualized with volcano plots, heatmaps, and enrichment plots to illustrate differential expression patterns and functional enrichment outcomes in Rstudio v1.4.

#### Small RNAs identification and target gene prediction

During preprocessing of small RNA sequencing data, adapter sequences were removed using cutadapt v5.0 (Martin, 2011), and reads between 18 and 32 nt were kept to target canonical miRNA and piRNA types. To remove potential contaminants, reads were first aligned to an rRNA/tRNA database with Bowtie2 v2.2 (Langdon, 2015), and matching sequences were discarded. The remaining high-quality reads were then aligned to the *Drosophila* miRNA and piRNA database. miRNA and piRNA expression levels were quantified with Salmon v1.10 (Patro et al., 2017), followed by differential expression analysis between groups using DESeq2 v1.12 (Love et al., 2014). Differentially expressed miRNAs (DEmiRs) were identified based on the criteria of |log_2_FC| ≥ 1 and raw *p-*value < 0.05. Given the high diversity and noise profile of piRNA data, the Benjamini-Hochberg FDR correction was applied to strictly control false positives. Accordingly, differentially expressed piRNAs were identified based on the criteria of |log_2_FC| ≥ 1 and FDR adjusted *p-*value < 0.05.

To investigate the regulatory roles of miRNAs in gene expression, target genes of the DEmiRs were predicted using miRanda v1.1 (Enright et al., 2003) and TargetScan v7.2 (Agarwal et al., 2018). Only the target genes identified by both algorithms were considered reliable. These predicted target genes were then cross-checked with transcriptome differential expression results. Finally, GO enrichment analyses were conducted on the candidate target genes to identify their associated biological processes and signaling pathways. For predicting piRNA targeting of TEs, miRanda and RNAhybrid were used to scan the identified TE sequences, only the target TEs identified by both algorithms were considered reliable.

#### TE identification and piRNA–TE association analysis

For analyzing transposable element (TE) expression in reproductive tissues, a *de novo Drosophila* repeat sequence library was initially created using RepeatModeler v2.0 (Flynn et al., 2020). Nucleotide sequences were then compared to both the Dfam database and the *de novo Drosophila* repeat library using RMBlast v2.10 and RepeatMasker v4.0 (Tarailo-Graovac and Chen, 2009). The TE classification was further refined with TEsorter v1.4. (Zhang et al., 2022) based on the RepeatMasker results. Transcriptome reads from reproductive tissues were aligned to the TE sequences, and TE expression levels were quantified with Salmon v1.10 (Patro et al., 2017). Differentially expressed TEs were identified using DESeq2 v1.12 (Love et al., 2014), with thresholds set at |log_2_FC| ≥ 1 and raw *p*-value < 0.05.

piRNA-generating clusters in the genome were identified using proTRAC v2.4 (Rosenkranz and Zischler, 2012) combined with custom scripts. To predict piRNA-TE interactions, miRanda v1.1 (Enright et al., 2003) and RNAhybrid v2.1 (Krüger and Rehmsmeier, 2006) were used on the identified TE sequences, and only overlapping predictions from both tools were considered reliable piRNA-targeted TEs. The resulting regulatory relationships were visualized using Cytoscape (https://cytoscape.org/). For significantly differentially expressed TEs, genome-wide alignment analyses were further performed to assess their copy number and genomic distribution. Genes located near these TEs were annotated and functionally analyzed to explore the potential roles of TEs in genomic stability and reproductive regulation.

### RT-qPCR

#### mRNA qPCR

To verify the reliability of the transcriptome sequencing results, several key genes showing significant differential expression in reproductive and immune-related pathways were selected for quantitative real-time PCR (qPCR) validation. Total RNA was extracted as described in the transcriptome sequencing section. cDNA was synthesized using the HiScript II RT SuperMix for qPCR (Vazyme, Nanjing, China). Primer sequences used for qPCR are listed in Supplementary Table S1. qPCR reactions were performed using the ChamQ Universal SYBR qPCR Master Mix (Vazyme) on a QuantStudio™ 5 Real-Time PCR System (Thermo Fisher Scientific). The amplification conditions were as follows: 95 °C for 30 s for initial denaturation, followed by 40 cycles of 95 °C for 10 s and 60 °C for 30 s. Each sample was run in triplicate, and *actin5C* served as the internal reference gene. Relative gene expression levels were calculated using the 2^−^ΔΔCt method and compared with RNA-seq expression trends for consistency.

#### miRNA stem-loop qPCR

For miRNAs that exhibit the most significant differential expression between males and females, specific stem-loop reverse transcription and qPCR primers were designed using the Vazyme miRNA Primer Design Tool (primer sequences are listed in Supplementary Table S1). cDNA synthesis was carried out with the Vazyme miRNA 1st Strand cDNA Synthesis Kit (by stem-loop), followed by amplification using the miRNA Unimodal SYBR qPCR Master Mix (Vazyme). Relative quantification was determined using the same 2^−^ΔΔCt method described for mRNA qPCR.

### Backcross experiments

To systematically evaluate the reproductive fitness of the F1 generation and determine whether the observed molecular dysregulation translates into functional fertility defects, backcrosses were performed between F1 progeny and their parental lines as follows: F1 males × T females and F1 females × W males. All backcross experiments used 1-day-old virgin flies. Virgin females were first placed individually in six-well plates and allowed to acclimate for 30 min, after which a single male was introduced. Once successful copulation was observed, the male was removed, and the mated female was transferred to a grape juice–agar plate coated with yeast paste for oviposition over 24 h. Eggs were then incubated at 25 °C, and the embryo hatch rate was recorded. Each cross type was replicated 10 times. Hatch rates were compared among F1 male × T female, F1 female × W male, *Wolbachia*-infected males and females, and *Wolbachia*-uninfected males and females.

### Statistical analysis

All qPCR experiments were performed with three independent biological replicates (*n* = 3). Data were presented as the mean ± standard error of the mean (SEM) for bar graphs, or as violin plots representing the data distribution. Statistical significance between the F1 offspring and their respective parental controls was determined using an unpaired two-tailed Student's *t*-test using GraphPad Prism 8.0 software. *p*-values < 0.05 were considered statistically significant (^*^p < 0.05, ^**^p < 0.01, ^***^p < 0.001, ^****^p < 0.0001).

## Result

### Immune-related genes were highly upregulated in the male offspring

Based on genome-wide comparisons, differential expression analysis was conducted using DESeq2 to compare gene expression profiles between offspring and their respective parental lines. In females, the F1 offspring showed gene expression patterns more similar to the maternal parent, while in males, the F1 offspring exhibited a notably higher number of differentially expressed genes compared to their parental lines (Figure 1B). A total of 126 differentially expressed genes (DEGs) (|log_2_FoldChange| ≥ 1, *p*-value < 0.01) were identified between F1 females and maternal controls (Figure 1C, Supplementary Table S2), whereas 278 DEGs (|log_2_FoldChange| ≥ 1, *p*-value < 0.01) were found between F1 males and paternal controls (Figure 1D, Supplementary Table S3). Functional enrichment analysis revealed distinct biological processes between the sexes. In females, DEGs were mainly enriched in pathways related to carbohydrate metabolism and negative regulation of immune function, indicating a decrease in immune activity in F1 females (Figure 1E). Conversely, in F1 males, DEGs were significantly enriched in innate immune pathways, with many immune- and detoxification-related genes strongly upregulated (Figure 1F). Additionally, numerous long non-coding RNAs (lncRNAs) and antisense RNAs (asRNAs) were differentially expressed in males, suggesting potential roles in post-transcriptional regulation.

Consistent with these findings, gene set enrichment analysis further showed that metabolic pathways related to detoxification, vitelline membrane and eggshell formation, egg activation, fertilization, and detoxification of zinc ions were significantly upregulated in the F1 male offspring. Conversely, pathways involved in stimulus detection, chemical sensory perception, sperm motility, and miRNA-mediated post-transcriptional gene silencing were notably downregulated, suggesting that sensory and sperm functions, as well as miRNA regulation, were suppressed in the F1 males (Figure 1G).

### Differential miRNA expression and miRNA-mRNA regulatory networks between F1 offspring and parental lines

**Figure 2:**
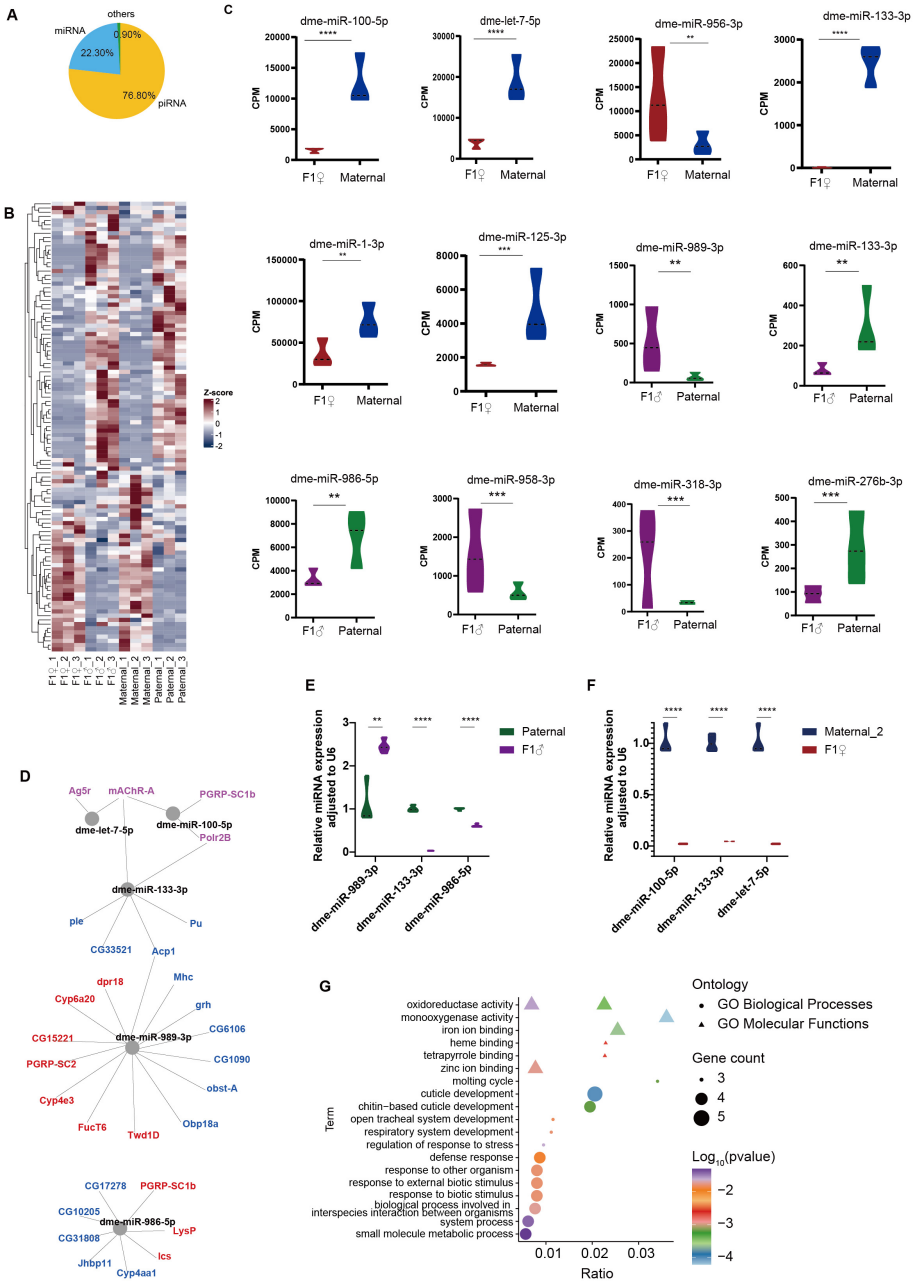
miRNA expression in reproductive tissues. **(A)** Composition of small RNA types in reproductive tissues. **(B)** Heatmap showing relative miRNA expression across all samples. Color scale represents *Z*-score normalized CPM values (Red: high expression; Blue: low expression). **(C)** Violin plot illustrating differential expression of miRNAs between groups CPM: Counts Per Million. **(D)** Predicted miRNA target genes. Purple indicates genes highly expressed in F1 females, red indicates genes highly expressed in F1 males, and blue indicates genes with low expression in F1 males. **(E)** qPCR validation of differentially expressed miRNAs between F1 males and paternal controls, using SnRNA U6 as an internal reference. **(F)** qPCR validation of differentially expressed miRNAs between F1 females and maternal controls, using SnRNA U6 as an internal reference. **(G)** GO enrichment analysis of target genes regulated by DEmiRs. Significance was determined by unpaired *t*-test (**p < 0.01, ***p < 0.001, ****p < 0.0001).

To explore the miRNA–mRNA regulatory networks further, small RNA-seq was conducted on the reproductive tissues of F1 offspring and parental lines. After filtering out rDNA sequences, small RNA reads were classified, showing that the majority of small RNAs in the *Drosophila* reproductive system were piRNAs, followed by miRNAs (Figure 2A). Most miRNAs did not show significant differential expression between the parental generation and the F1 offspring (Figure 2B, Supplementary Tables S4, S5). When comparing F1 males to paternal lines, six miRNAs (dme-miR-989-3p, dme-miR-133-3p, dme-miR-986-5p, dme-miR-958-3p, dme-miR-318-3p, dme-miR-276b-3p) were differentially expressed (|log_2_FoldChange| ≥ 1, *p*-value < 0.05) (Figure 2C). Similarly, six miRNAs were differentially expressed between F1 females and maternal lines (dme-miR-100-5p, dme-let-7-5p, dme-miR-133-3p, dme-miR-125-5p, dme-miR-956-3p, dme-miR-1-3p) (Figure 2C). For the most differentially expressed miRNAs between groups, target genes of these miRNAs were predicted and intersected with DEGs to identify potential regulatory interactions (Figure 2D). And key miRNAs were further validated by qPCR to measure their expression levels in F1 offspring relative to parental lines (Figures 2E, F).

GO enrichment analysis of miRNA-regulated DEGs revealed significant enrichment in pathways including peptidoglycan muralytic activity, immune defense response, cuticle development, and small-molecule metabolism (Figure 2G). Reproduction-related genes such as *Pu, ple, Acps*, and *grh* were significantly downregulated and immune-related genes *PGRP-SC1b* and *PGRP-SC2* were upregulated in F1 males. qPCR also confirmed the differential expression of these genes between the F1 offsprings and the parental lines. The qPCR validation results for these genes are shown in Supplementary Figure S1.

### Differential expression of transposable elements in F1 offspring and their chromosomal distribution

**Figure 3:**
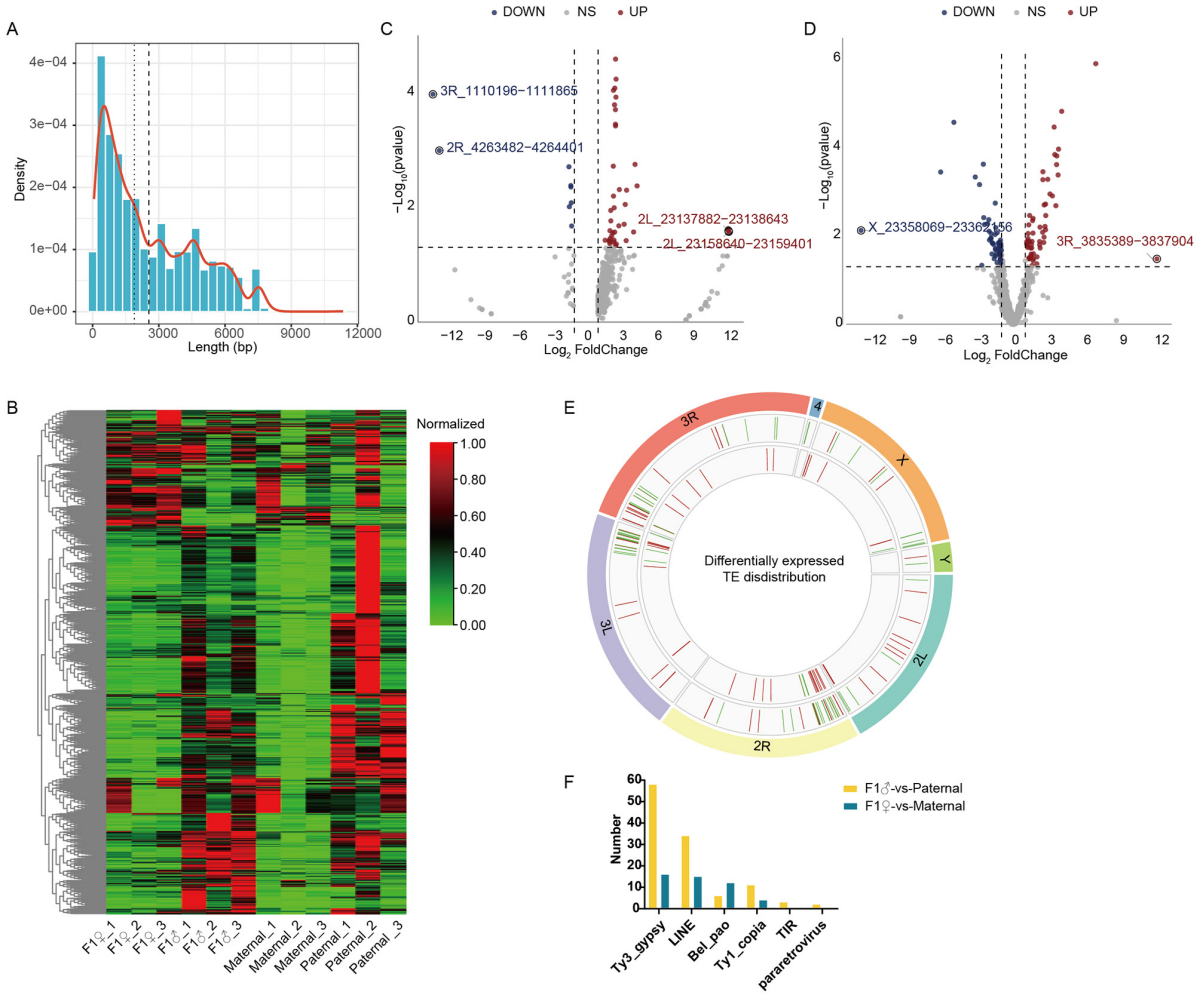
TE expression in *Drosophila* reproductive tissues. **(A)** Length distribution of *de novo* identified TEs in the *Drosophila* genome. **(B)** Heatmap showing the relative expression of all TEs across samples. Expression levels were normalized to a scale of 0 to 1 (Min–Max normalization) as indicated by the color key in the upper right (Red: high expression; Green: low expression). **(C)** Volcano plot of differentially expressed TEs between F1 females and maternal lines. **(D)** Volcano plot of differentially expressed TEs between F1 males and paternal lines. **(E)** Circos plot illustrating the chromosomal distribution of differentially expressed TEs. From outer to inner circles: the first circle represents chromosomes, the second shows the distribution of differentially expressed TEs between F1 females and maternal lines, and the third depicts the distribution of differentially expressed TEs between F1 males and paternal lines. **(F)** Annotation and classification of differentially expressed TEs between groups.

Small RNA-seq also showed high piRNA levels in *Drosophila* reproductive tissues. We first identified TE sequences in the *Drosophila* genome using RepeatMasker and TEsorter, then quantified the expression of each TE using transcriptome data. Among all identified TEs, sequence lengths were mainly distributed between 300 and 7,000 bp (Figure 3A). TE expression was higher in male testes and relatively lower in ovaries (Figure 3B). Differential expression analysis between F1 offspring and parental lines revealed that in F1 females vs. maternal females, only 47 TEs showed significant differential expression (|log_2_FoldChange| ≥ 1, *p*-value < 0.05), including 8 downregulated and 29 upregulated TEs (Figure 3C, Supplementary Table S6). In F1 males vs. paternals, 106 TEs were differentially expressed, with 48 downregulated and 58 upregulated (Figure 3D, Supplementary Table S7). We further analyzed the chromosomal distribution of these differentially expressed TEs. They were mainly enriched on chromosomes 2R, 3L, and 3R (Figure 3E), and mostly belonged to the Ty3_gypsy and LINE families (Figure 3F).

### The regulation of piRNA-TE was imbalanced in the male offspring

**Figure 4:**
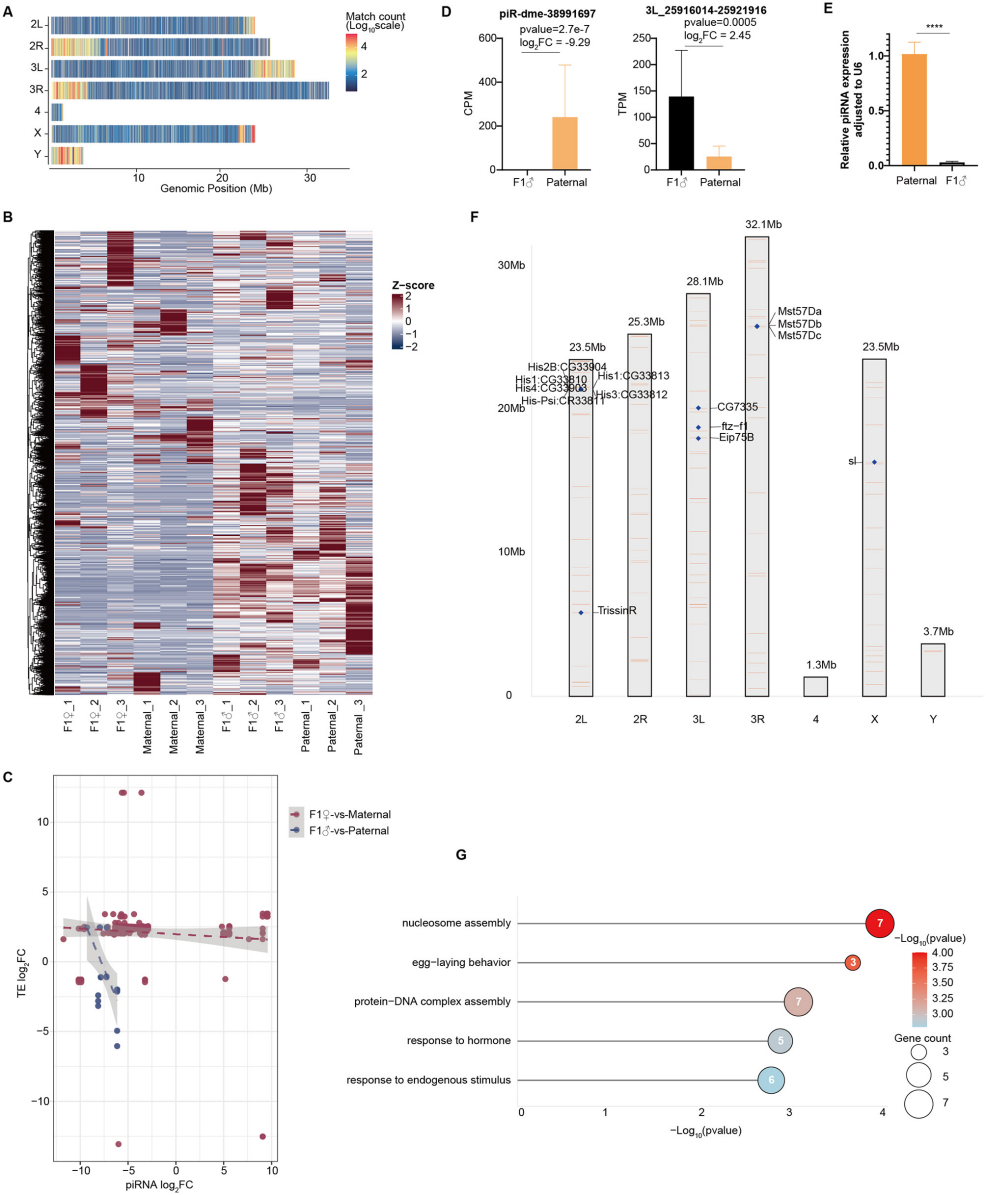
Regulatory relationship between piRNAs and TEs in reproductive tissues. **(A)** Identification of piRNA clusters. **(B)** Heatmap displaying relative expression levels of all piRNAs across samples, with CPM values normalized using *Z*-scores. **(C)** Correlation analysis of fold changes between piRNAs and their target TEs. **(D)** Differential expression of piR-dme-38991697 and its target TE between F1 males and paternal flies. **(E)** qPCR validation of piR-dme-38991697 expression in parental and offspring samples. Data are presented as mean ± SEM. Significance was determined by unpaired *t*-test (****p < 0.0001). **(F)** Genomic distribution and neighboring genes of TE 3L_25916014–25921916 across the *Drosophila* genome. **(G)** Functional enrichment analysis of genes near the genomic copies of TE 3L_25916014–25921916.

We then examined how piRNAs regulate transposable elements (TEs) in the *Drosophila* reproductive system. Due to the high expression of piRNAs in reproductive tissues, we initially analyzed the genomic origins of piRNAs (piRNA clusters). Genome-wide mapping showed that piRNAs are mainly generated at chromosomal ends, especially in telomeric regions (Figure 4A), and piRNAs exhibited higher relative expression in male testes (Figure 4B, Supplementary Figure S2). Potential TE targets of piRNAs were identified using Blastn by searching for seed matches (7-mer) with one mismatch allowed. Correlation analyses between piRNA expression and the expression of their targeted TEs revealed no significant correlation in ovaries but a clear negative correlation in testes (Figure 4C). Differential expression analysis indicated that 302 piRNAs were differentially expressed between F1 females and maternals, while only 32 piRNAs showed differential expression between F1 males and paternals (Supplementary Tables S8, S9).

Given the higher expression of both piRNAs and TEs in male testes and the clear negative regulation, we focused on the most differentially expressed piRNAs in males. piR-dme-38991697 was downregulated by nearly 1,000-fold in F1 males, and target prediction indicated that its corresponding TE was upregulated about six-fold (Figures 4D, E). The TE 3L_25916014-25921916, with a length of 5,902 bp, was further examined for genomic copy number using Blastn with a minimum match length of 4,000 bp and an E-value threshold of 0.001. This TE was present in 121 copies across all chromosomes except chromosome 4 (Figure 4F). Frequent TE insertions and transpositions may disrupt neighboring gene functions. We searched for all genes within a 10 kb region surrounding each of the 121 TE copies and performed functional annotation. Many genes related to chromosome assembly and responses to external stimulite, including multiple histone genes, were found near these TEs copies (Figure 4G), suggesting potential impacts of TE activity on genomic stability and gene regulation.

To evaluate the reproductive capacity of the CI offspring, backcross experiments were performed. The results showed that the hatching rate of the F1 male × T female crosses was significantly lower than that of the F1 female × W male crosses (Supplementary Figure S3), indicating that the reproductive capacity of male offspring was markedly reduced compared with that of female offspring.

## Discussion

CI caused by *Wolbachia* has long been a major focus in studies of symbiosis and reproductive biology (Hochstrasser, 2023). Earlier research mainly examined how *Wolbachia* infection affects males, with limited understanding of the molecular and reproductive effects on offspring resulting from CI cross. It remains unclear whether *Wolbachia*-induced male infertility impacts the reproductive capacity of the next generation. Our transcriptomic and small RNA sequencing showed that F1 offspring from CI crosses display notable sex-biased responses in reproductive tissues (Figures 1B, 2B). In male offspring, genes involved in immune and detoxification pathways were significantly upregulated, whereas those related to sperm function were mostly downregulated (Figures 1F, G). It is important to note that these F1 males are survivors from a high-mortality cohort, and while immune activation may partially reflect a stress response to early developmental challenges (Sheehan et al., 2020). However, the specific downregulation of sperm-related genes points to a CI-induced dysregulation passed to the offspring, rather than a consequence of generalized somatic damage during embryonic development. Additionally, disruptions in the small RNA regulatory network may decrease fertility in the offspring. Notably, although fewer differentially expressed piRNAs were identified in F1 males compared to females, the stronger negative correlation between these piRNAs and their predicted target TEs (Figure 4C) suggests a functionally significant dysregulation of the piRNA pathway in the testes. Our findings indicated that *Wolbachia* impairs male reproductive function in CI offspring through complex molecular pathways, including immune and metabolic regulation, miRNA control, and the piRNA-TE axis.

Considering that this study compares uninfected *Wolbachia* F1 males with their *Wolbachia*-infected paternal controls, there is an issue regarding different *Wolbachia* infection statuses. We compared the results of this experiment with previous transcriptomic results of *Wolbachia*-infected and uninfected males. Interestingly, we found that although *Wolbachia*-infected males in previous experiments showed significant upregulation of immune genes (Zheng et al., 2011b; Dou et al., 2021), the uninfected F1 males in this experiment showed significant upregulation of immune genes compared with their paternal lines. These two completely opposite regulations regarding immune genes illustrated that the effects observed between the F1 generation and the parental lines were largely regulated by CI. Immune activation induced by CI may therefore contribute to reduced male reproductive performance through an immune-reproduction trade-off. Previous studies have shown that *Wolbachia* infection can activate host innate immune responses or induce oxidative stress, enhancing resistance to pathogens but often at the cost of diverting energy and resources away from reproduction (She et al., 2024; Mushtaq et al., 2024; Critchlow et al., 2024). In this study, the increased expression of immune-related genes and the reduced activity of reproduction-related pathways in the male offspring support the immune-reproduction trade-off hypothesis.

Our small RNA sequencing identified a distinct dysregulation of the miRNA profile in F1 offspring (Figure 2B). This finding aligned with the growing consensus that miRNA-mediated gene regulation is a primary mechanism through which endosymbionts manipulate host reproductive and physiological processes (Dai et al., 2024; Lu et al., 2024). Previous studies showed that *Wolbachia* infection in *Drosophila* upregulates a specific miRNA, nov-miR-12, which directly suppresses the expression of *pipsqueak* (*psq*), a gene essential for embryonic patterning (Zheng et al., 2019). The downregulation of *psq* was sufficient to recapitulate key CI phenotypes, including chromatin bridging and asynchronous nuclear division (Zheng et al., 2019). Similar regulatory strategies have been observed in other insect systems; for instance, *Wolbachia* upregulates aae-miR-34-3p in *Aedes aegypti* to boost antiviral immunity (She et al., 2024), and juvenile hormone modulates miR-2/miR-109 in *Diaphorina citri* to improve fecundity during infection (Nian et al., 2024). Consistent with these precedents, although we identified a limited number of differentially expressed miRNAs in F1 males (Figure 2C), their predicted targets were critically involved in key biological processes (Figures 2D, G). Notably, dme-miR-989-3p emerged as a central regulatory hub interacting with multiple gene clusters that showed distinct expression patterns in F1 males. Specifically, this miRNA targeted several downregulated genes essential for reproductive success and structural integrity, including *Acp*s, *ple, grh, Pu*, and *obst-A* (Pesch et al., 2015; Ahmed and Vogel, 2020). Conversely, immune and detoxification regulators, including *PGRP-SC2* and *PGRP-SC1b* and Cytochrome P450s (e.g., *Cyp6a20, Cyp4e3*), were found to be upregulated (Persson et al., 2007). The dysregulation of these miRNA-mRNA axes, involving the suppression of fertility-associated genes together with the activation of immune pathways, suggested that *Wolbachia*-induced small RNA alterations may function as persistent intergenerational determinants of host fitness.

In addition to miRNAs, we observed a severe disruption of the piRNA pathway in F1 reproductive tissues, characterized by the specific downregulation of piRNAs and the upregulation of TEs (Figure 4). Studies have shown that piRNAs are mainly expressed in reproductive tissues and form PIWI–piRNA silencing complexes (PISC) to control transposable element (TE) expression. Imbalances in the piRNA-TE regulatory system can cause hybrid infertility or reproductive issues (Romero-Soriano et al., 2017). The disruption of the piRNA-TE regulatory axis is a crucial mechanism connecting small RNA regulation to genomic stability. Growing evidence showed that transposon instability is closely linked to male reproductive infertility (Stallmeyer et al., 2024; Wei et al., 2024). In both humans and mice, biallelic mutations in key piRNA pathway genes such as PIWIL1 and GTSF1 have been found to impair piRNA biogenesis, leading to the derepression of transposable elements (TEs) like LINE1. This TE activation has been demonstrated to cause defective spermatogenesis or germ cell loss (Stallmeyer et al., 2024). Similarly, in *Drosophila*, piRNAs are the main regulators of TEs within the reproductive system, ensuring genomic integrity and proper gametogenesis (Chen et al., 2021). Overall, these findings highlight that the piRNA pathway is essential for silencing transposon activity and maintaining germline stability across species.

Our analysis revealed significant upregulation of TEs in the testes of CI hybrid male offspring (Figures 3B, D). In contrast, most TEs remained largely unchanged in CI hybrid females, indicating a more pronounced disruptive effect of hybridization in males, consistent with the established model of piRNA-mediated TE suppression (Lawlor et al., 2021). Notably, a specific piRNA (piR-dme-38991697) was dramatically downregulated in F1 males, along with an approximately 6-fold increase in its target TE (Figures 4D, E), highlighting its key role in suppressing specific transposon activity. Genome-wide mapping revealed that this TE was distributed across multiple chromosomes in numerous copies (Figure 4F), with nearby genes enriched for chromatin organization and stress-response functions, including several histone genes (Figure 4G). These findings suggested that frequent TE insertions may not only disrupt local gene function but also alter chromatin structure and transcriptional homeostasis, ultimately threatening germline genomic stability. Recent studies further indicate that *Wolbachia* infection can modulate host TE activity (Eugénio et al., 2023), supporting our hypothesis that an imbalance in piRNA-TE regulation constitutes a molecular pathway through which *Wolbachia* impairs male reproductive functions. Together, these results highlight piRNA-mediated TE repression as a core mechanism for maintaining germline genome integrity and facilitating population genetic stability and adaptive evolution.

*Wolbachia* shows clear sex-biased patterns on its hosts. As a maternally transmitted endosymbiont, *Wolbachia*'s transmission and survival strategies are mainly aim to enhance the fitness and reproduction of female host, while often having harmful effects on males, such as causing male embryo death (male killing) (Arai et al., 2025; Hornett and Hurst, 2023). Consistent with this pattern, we also observed distinct transcriptional responses between the sexes. Female offspring maintained a transcriptomic profile highly similar to their maternal lines, showing minimal transcriptomic changes and a significant increase in genes involved in immune suppression. In contrast, the male offspring showed more extensive transcriptional changes, with notable upregulation of many immune and detoxification-related genes (Figure 1). Additionally, analysis of small RNAs indicated that male-biased differentially expressed miRNAs regulated more DEGs (Figure 2B), and the testis had higher TE expression compared to the ovary. A strong negative correlation between piRNAs and their target TEs was found in testes, but this relationship was not significant in ovaries. Overall, the data support a model in which *Wolbachia* infection causes male-biased reprogramming of the host's molecular landscape. In males, this reprogramming involved the simultaneous activation of immune pathways and a major disruption of the TE-piRNA regulatory axis.

## Conclusion

This study used combined transcriptome and small RNA sequencing analyses to clarify the molecular mechanisms behind differences between CI offspring and their parental lines. Results showed that the male offspring had significant increases in immune and detoxification-related pathways in reproductive tissues, while key pathways involved in spermatogenesis, energy metabolism, and small RNA regulation were largely suppressed. This pattern indicates an immune–reproduction trade-off. Additionally, differentially expressed miRNAs were identified as key regulators of genes involved in reproduction, development, and immunity. Notably, an imbalance in the piRNA-TE axis was observed in F1 male testes, characterized by the downregulation of piR-dme-38991697 and concomitant upregulation of its target transposable element, underscoring its critical role in maintaining genomic stability and reproductive health. In contrast, the female offspring showed minimal molecular differences from their mother. Overall, these findings reveal that *Wolbachia* causes complex molecular reprogramming, with more harmful effects mainly in male offspring from CI cross, offering new insights into the molecular basis of *Wolbachia*-induced cytoplasmic incompatibility. Specifically, we demonstrate the intergenerational effects (parent to F1) of CI; whether these dysregulated states persist beyond the F1 generation would be an intriguing subject for future study.

## Data Availability

The data presented in this study are publicly available. The data can be found at: https://www.ncbi.nlm.nih.gov/sra, accession PRJNA1346818.
