# Supplementary material for: Wolbachia-induced cytoplasmic incompatibility triggers intergenerational dysregulation of the small RNA regulatory network in offspring
Source: Front Microbiol. 2026 Mar 5;17:1764569. doi: 10.3389/fmicb.2026.1764569 (PMC13001649; doi:10.3389/fmicb.2026.1764569)
Supplement: Supplementary file 2 [file Data_Sheet_1.docx]

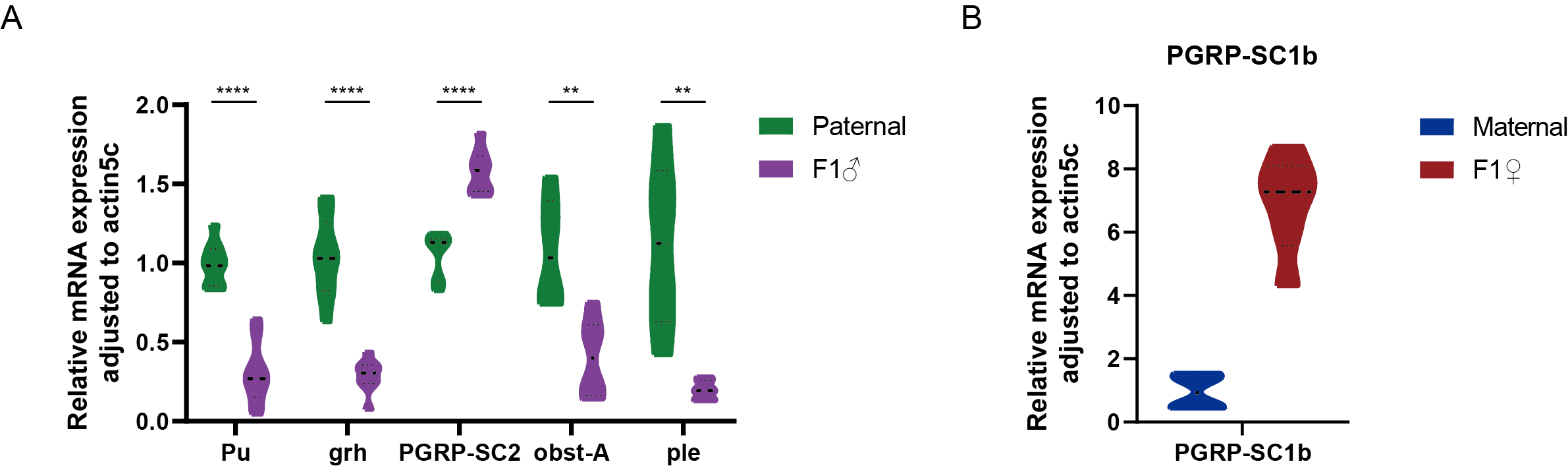


Figure S1. (A) qPCR validation of DEGs regulated by DEmiRNAs in males. (B) qPCR validation of DEGs regulated by DEmiRNAs in females. Significance was determined by unpaired t-test (*: *p* < 0.05, **: *p* < 0.01, ***: *p* < 0.001, ****: *p* < 0.0001).


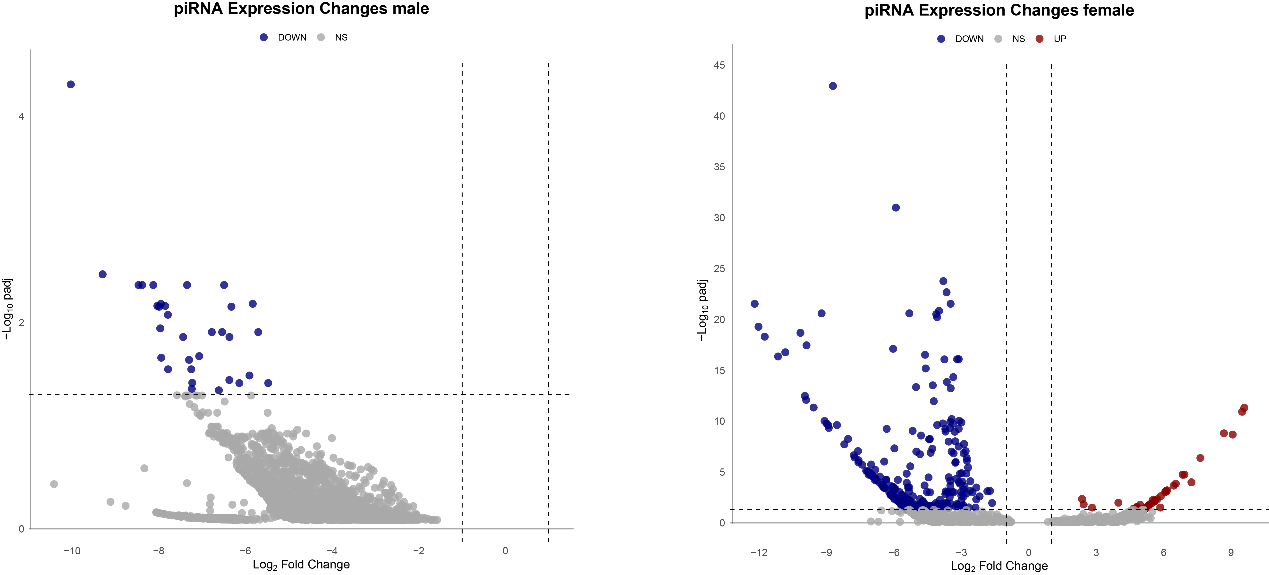


Figure S2. Volcano plot of differentially expressed TEs in males and females.


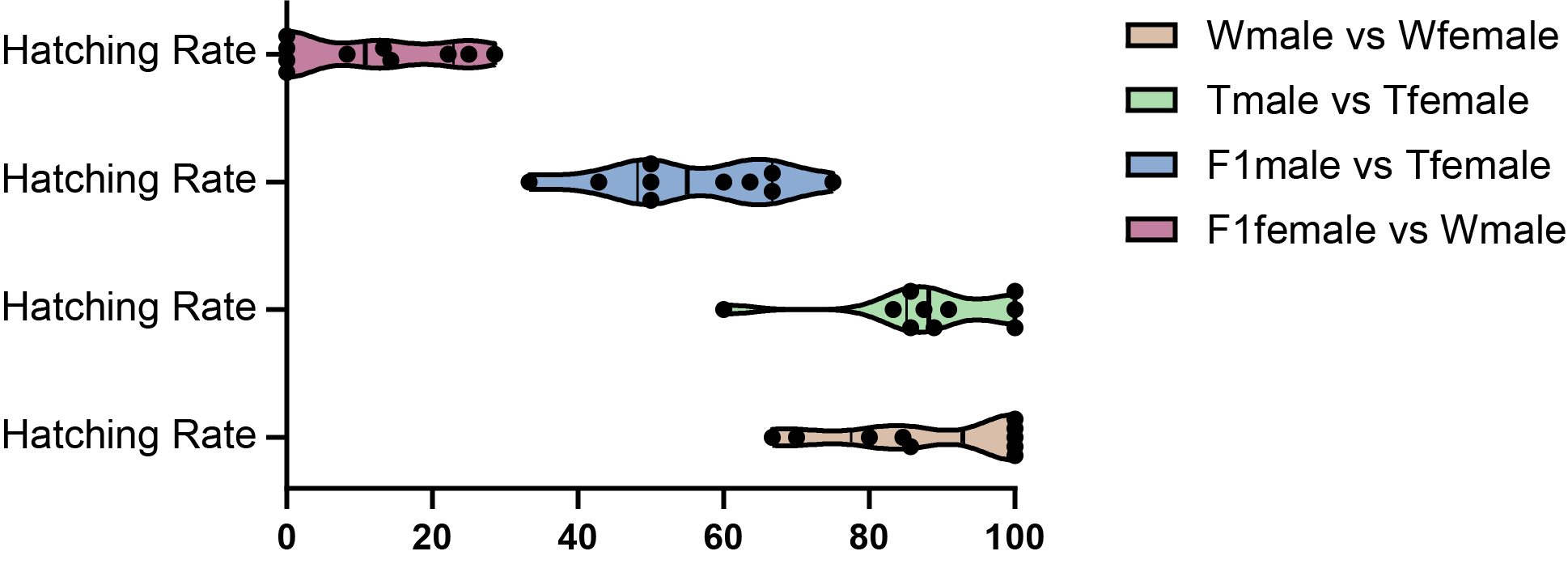


Figure S3 Hatching rate of the backcross experiment.
